# Long non-coding RNA *HOTAIR* polymorphism and susceptibility to cancer: an updated meta-analysis

**DOI:** 10.1186/s12199-018-0697-0

**Published:** 2018-02-20

**Authors:** Juan Li, Zhigang Cui, Hang Li, Xiaoting Lv, Min Gao, Zitai Yang, Yanhong Bi, Baosen Zhou, Zhihua Yin

**Affiliations:** 1 Department of Epidemiology, School of Public Health, China Medical University, Shenyang, 110122 China; 2 Key Laboratory of Cancer Etiology and Intervention, University of Liaoning Province, No. 77 Puhe Road, Shenyang North New Area, Shenyang, 110122 China; 3 School of Nursing, China Medical University, Shenyang, 110122 China

**Keywords:** Cancer, HOTAIR, LncRNAs, Polymorphism, Susceptibility

## Abstract

**Background:**

An increasing number of publications are drawing attention to the associations between six common polymorphisms in HOX transcript anti-sense RNA (HOTAIR) and the risk of cancers, while these results have been controversial and inconsistent. We conducted an up-to-date meta-analysis to pool eligible studies and to further explore the possible relationships between HOTAIR polymorphisms (rs920778, rs7958904, rs12826786, 4,759,314, rs874945, and rs1899663) and cancer risk.

**Methods:**

A systematic retrieval was conducted up to 1 July 2017 in the PubMed, Web of Science, and CNKI databases. Eighteen eligible publications including 45 case-control studies with 58,601subjects were enrolled for assessing the associations between the 6 polymorphisms in HOTAIR and cancer risk. Pooled odds ratios (ORs) with 95% confidence intervals (CIs) were analyzed to reveal the polymorphisms and susceptibility to cancer. All the statistical analyses were performed using STATA 11.0 software.

**Results:**

The pooled analyses detected significant associations between the rs920778 polymorphism and increased susceptibility to cancer in recessive, dominant, allelic, homozygous, and heterozygous models. For the rs7958904 polymorphism, we obtained the polymorphism significantly decreased susceptibility to overall cancer risk among five genetic models rather than recessive and homozygous models. For the rs12826786 polymorphism, we identified it significantly increased susceptibility to cancer risk in all genetic models rather than heterozygous models. However, no significant association was found between the rs1899663, rs874945, and rs4759314 polymorphisms and susceptibility of cancer.

**Conclusion:**

These findings of the meta-analysis suggest that HOTAIR polymorphism may contribute to cancer susceptibility.

**Electronic supplementary material:**

The online version of this article (10.1186/s12199-018-0697-0) contains supplementary material, which is available to authorized users.

## Background

Long non-coding RNAs (lncRNAs) are a class of particular no-coding RNA molecules with lengths of more than 200 nucleotides (nt), which are mainly produced by RNA polymerase II (RNA pol II) transcription, lack of protein-encoding function, and an open reading frame [1, 2]. Several studies have demonstrated that lncRNAs not only act as intermediaries between DNA and proteins, but also as a kind of crucial substances with affecting cell function involved in the epigenetic modification, transcription, and post-transcription process to regulate gene expression ultimately [3, 4].Therefore, aberrant regulation of lncRNAs is often associated with a variety of diseases, especially cancers. In recent years, the whole genome cancer mutation analysis gradually identified lncRNAs properties, focusing on the complex regulation of lncRNA transcription how to contribute many important cancer phenotypes. Schmitt et al. revealed molecular mechanisms of the lncRNA interacting with DNA, RNA, and protein, and also generalized lncRNAs played vital role in intracellular signal transduction networks in the carcinogenesis and progression of various cancers as drivers of the cancer phenotypes [5]. Some cancer-related lncRNAs could affect the development and progression of cancer by means of *p53*, polycomb repressive complex 2 (*PRC2*), and other signaling pathways. In p53 complex signaling pathways, Grossi et al. reported that some lncRNAs can directly or indirectly regulate p53 activity leading to upregulation or downregulation of *p53* expression [6]. In addition, to directly participate in the regulation of gene expression, lncRNAs also could compete with the same miRNA with other RNA transcripts as ceRNA (competing endogenous RNAs) to regulate expression of target genes at the posttranscriptional level [7].

HOX transcript anti-sense RNA (*HOTAIR*) is located on between the *HOXC11* and *HOXC12* coding region in the chromosome 12q13, which consists of five short exons and one long exon [8]. Significantly high expression of *HOTAIR* has been indicated in breast cancer [9], pancreatic cancer [10], liver cancer [11], colorectal cancer [12], lung cancer [13], and other malignant tumors, and with which tumor cells invasion and metastasis, tumor recurrence, and poor prognosis are closely related. It has been found that *HOTAIR* regulates the expression of genes by specifically binding different histone-modified complexes. As one of the regulators of oncogene transcription, *HOTAIR* remodels *PRC2* and desmethylase *LSD1* complexes by recruiting chromatin to cause demethylation of Histone *H3K27em3*, silence of *HOXD* gene expression, ultimately facilitate tumor proliferation or metastasis at the epigenetic level [14, 15]. *HOTAIR* also could form regulation network of ceRNAs with miRNA to participate in carcinogenesis of different cancers. Moreover, *HOTAIR* also acts as miRNA sponge in combination with miR-331-3p or miR-124 [16], leading to upregulation of *HER2* and other relevant genes and activation of *Akt* signaling pathway, which could simultaneously interact with *p53* pathway to promote proliferation, invasion, and metastasis of tumor cell [17]. In the final analysis, *HOTAIR* was intricately related to various cancers.

Several single nucleotide polymorphisms (SNPs) in *HOTAIR* are widely researched in cancer susceptibility, prognosis [18], and clinical outcome [19] and treatment response [20]. Recently, some published results have definitely shown the inconsistent and controversial associations of common SNPs (rs1899663, rs4759314, rs920778, rs874945, rs7958904, and rs12826786) in *HOTAIR* with the risk of various cancers including digestive cancers [21–29], estrogen-dependent cancers [18, 19, 30–33], papillary thyroid carcinoma [34], glioma [35], pancreatic cancer [36], prostate cancer [37], and osteosarcoma [38]. Herein, we indispensably conducted a meta-analysis to summarize all currently eligible case-control studies to more accurately elucidate the authentic associations between *HOTAIR* polymorphisms and cancer susceptibility, especially, for rs12826786 C>T, this is first meta-analysis to evaluate the relationship between the polymorphism and cancer risk in overall population.

## Methods

### Literature search

Eligible literatures were systematically retrieved in several authoritative databases including PubMed, Web of Science, and CNKI databases to search comprehensive and systematic publications up to 1 July 2017, with the following keywords including “long non-coding RNA *HOTAIR* OR lncRNA *HOTAIR* OR *HOTAIR*,” “polymorphism OR variation OR mutation,” and “cancer OR carcinoma OR tumor OR malignancy OR neoplasm OR lymphoma OR leukemia.” Moreover, this study further retrieves the references lists of eligible studies to guarantee that all qualified studies are included in the meta-analysis.

### Inclusion and exclusion criteria

Included criteria of eligible studies were as follows: (a) studies estimating the associations between lncRNA *HOTAIR* polymorphisms or genetic variations and the risk or susceptibility of various cancers, (b) case-control designed studies with genotypes distribution of controls followed Hardy–Weinberg equilibrium, (c) studies with available or adequate genotype data for calculation of the odds ratio (OR) with 95% confidence intervals (95% CIs). The main criteria for excluding unqualified researches were as follows: (1) did not focus on cancer risk or susceptibility, (2) did not involve the several *HOTAIR* SNPs (rs920778, rs4759314, rs1899663, rs7958904, rs874945, or rs12826786), (3) did not present the sufficient genotype frequency data of cases and controls. Ultimately, a total of 18 articles consist of 14,119 cases and 16,295 controls were included in this meta-analysis (presented in Table 1).

**Table 1:** Characteristics of studies on HOTAIR polymorphism and cancer risk

First author	Year	Country	Ethnicity	Source of control	Genotyping methods	Type of cancers	Case	Control	Included in meta-analysis
Zhang [21]	2014	china	Asian	PB	RFLP	ESCC	1000	1000	Yes
Bayram [33]	2015	Turkey	Caucasian	HB	TaqMan	BC	123	122	Yes
Bayram [28]	2015	Turkey	Caucasian	HB	TaqMan	GC	104	209	Yes
Du [29]	2015	china	Asian	HB	Taqman	GC	753	1057	Yes
Guo [25]	2015	china	Asian	PB	RFLP	GCA	515	654	Yes
Xue [22]	2015	china	Asian	HB	Taqman	CRC	1147	1203	Yes
Yan [31]	2015	china	Asian	PB	RFLP	BC	502	504	Yes
Bayram [19]	2016	Turkey	Caucasian	HB	TaqMan	BC	123	122	Yes
Guo [32]	2016	china	Asian	HB	MALDI-TOF-MS	CC	510	713	Yes
Pan [26]	2016	china	Asian	PB	RFLP	GC	500	1000	Yes
Qiu [18]	2016	china	Asian	HB	Taqman	Ovarian cancer	190	380	No
Qiu [24]	2016	china	Asian	NA	Taqman	CC	215	430	No
Wu [30]	2016	china	Asian	NA	MALDI-TOF-MS	EOC	1000	1000	Yes
Zhou [38]	2016	china	Asian	HB	MALDI-TOF-MS	Osteosarcoma	500	500	Yes
Zhu [34]	2016	china	Asian	NA	RFLP	PTC	1000	1000	Yes
Hu [36]	2017	china	Asian	PB	TaqMan	Pancreatic cancer	416	416	Yes
Jin [27]	2017	china	Asian	HB	TaqMan	CC	1174	1304	Yes
Taheri [37]	2017	Iran	Caucasian	HB	ARMS-PCR	Prostate cancer	128	250	Yes
Ulger [23]	2017	Turkey	Caucasian	HB	TaqMan	GC	105	207	Yes
Xavier‑Magalhaes [35]	2017	Portugal	Caucasian	PB	RFLP	Glioma	177	199	Yes

*BC* Breast cancer, *CRC* Colorectal cancer, *CC* Cervical cancer, *ESCC* Esophageal squamous cell carcinoma, *GC* Gastric cancer, *GCA* Gastric cardia denocarcinoma, *PTC* Papillary thyroid carcinoma, *HB* Hospital-based, *PB* Population-based, *RFLP* restriction fragment length polymorphism, *MOLDI-TOF-MS* Matrix-Assisted Laser Desorption/ Ionization Time of Flight Mass Spectrometry

### Data extraction

Two investigators (L.J. and L.X.) independently gathered the following information from each qualified publication: the first author’s name, year, country, and ethnicity of eligible publication, type of neoplasm, source of control, genotyping method, numbers of cases and controls, distribution of genotype frequencies as well as allele for cases and controls, *P* value of Hardy–Weinberg equilibrium (HWE) of control group, adjusted factors in the statistical analysis for each publication, and assessment of article quality. Two investigators reached a consensus on the basis of discussion when they have different opinions.

### Quality score assessment

Two investigators individually assessed the quality of all included studies according to the Newcastle-Ottawa scale (NOS), and the scale totally comprises subject selection, comparability of cases, and controls as well as ascertainment of exposure.

### Statistical analysis

Chi-square test was conducted to evaluate whether genotype distribution of the control group follow HWE. *Q* test and *I*^2^ test were used to examine the heterogeneity between each study, and the random effect model is used to combine the relevant studies when *I*^2^ is greater than 50%, otherwise, using a fixed-effect model and merged odds ratio (OR), and 95% confidence interval (95% CI) to evaluate the relationship between cancer risk and *HOTAIR* polymorphisms. In the current study, sensitivity analysis is to evaluate whether a single study would impact the overall effect value of the integrated researches. Begg’s funnel plots and Egger’s test was performed to uncover underlying publication bias by means of Stata 11. All calculations of the present meta-analysis were operated with Stata 11. *P* < 0.05 was uniformly regarded as a significant difference.

## Results

### Characteristics of the published studies

**Fig. 1:**
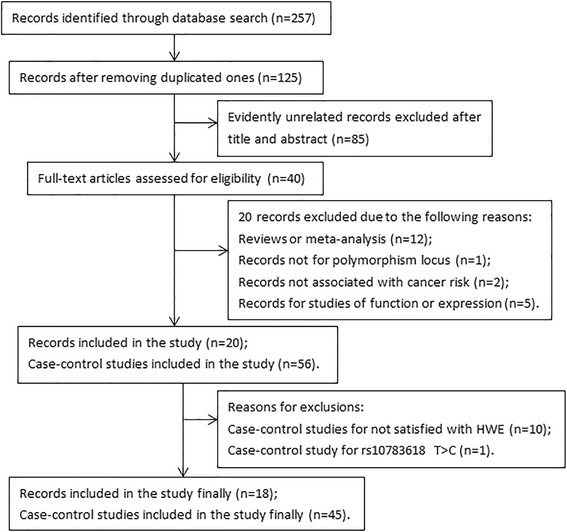
Flow diagram of articles identified with included and excluded criteria

The main characteristics of 20 articles on relationship between 6 *HOTAIR* SNPs and cancer risk were presented in Table 1, and genotype distribution information of the 6 polymorphisms about case-control studies is shown Additional file 1: Table S1 in detail. A total of 18 eligible publications including 45 case-control studies with 58,601 subjects (comprising 27,016 cases and 31,585 controls) met the inclusion criteria and 6 SNPs were involved in the meta-analysis. Of the 45 case-control studies included in meta-analysis, the *HOTAIR* rs4759314 in 13 studies [21, 22, 25–27, 29, 31, 32, 34, 36, 37], rs920778 in 12 studies [21, 26, 28, 31–35], rs7958904 in 6 studies [22, 27, 29, 30, 38], rs874945 in 3 studies [22, 27, 29] rs1899663 in 6 studies [21, 26, 31, 32, 34, 37], and rs12826786 in 5 studies [19, 23, 25, 35, 37] were analyzed, respectively. Of the all case-control studies listed in Additional file 1: Table S1, 11 studies presented a significant deviation from HWE (2 studies on rs4759314 [30, 38], 4 on rs920778 [18, 24, 26], 1 study on rs7958904 [38], 1 study on rs12826786 [25], and 3 on rs874945 [30, 38]). The detailed flow diagram of the articles selection process is presented in Fig. 1. Genotyping method involved in PCR-RFLP, MALDI-TOF-MS, ARMS-PCR, and TaqMan methods. All of these studies mainly focused on the study of female-related cancers and digestive system cancer.

### Analysis of quantitative synthesis

#### Rs920778, rs12826786, and rs7958904 polymorphism and cancer susceptibility

There were a total of 12 eligible case-control studies with 6187 cases and 6897 controls that focused on the associations of rs920778 C>T and cancer risks after excluding the 4 studies (Qiu-a, b, and c and Pan-a) that their controls did not meet HWE as shown in Table 1. The pooled results for the association between rs920778(C>T) polymorphism and cancer risk are presented in Table 2. The pooled analyses indicated that rs920778 polymorphism significantly increased with susceptibility to overall cancer in all five genetic (CTvs.CC: OR = 1.298, 95% CI = 1.203–1.400, *I*^2^ = 13.0%; TT vs.CC: OR = 2.289, 95% CI = 1.815–2.887, *I*^2^ = 54.3%; CT + TT vs. CC: OR = 1.422, 95% CI = 1.322–1.529, *I*^2^ = 31.10%; TT vs. CT + CC: OR = 2.028, 95% CI = 1.641–2.506, *I*^2^ = 57.90%; T vs. C: OR = 1.414, 95% CI = 1.336–1.497, *I*^2^ = 48.2%) models. Additionally, the similar relations were obtained both in cancer type (including estrogen-dependent cancers and digestive cancers) and source of controls subgroup. The heterogeneity was conspicuously reduced in both Caucasian and Chinese population according to the results of Table 2 presented, and, in particular, this significant risk was observed in the Chinese population except Caucasian, and the decreased heterogeneity was also observed in the other subgroup.

**Table 2:** Results of meta-analysis the relationship between HOTAIR SNPs and cancer risk

Subgroup	n	Heterozygote vs. Wild-type			Mutation homozygote vs. wild-type			Dominant model			Recessive model			Allelic model		
		OR(95%CI)	P	I ^2^(%)	OR(95%CI)	P	I^2^ (%)	OR(95%CI)	P	I^2^ (%)	OR(95%CI)	P	I^2^ (%)	OR(95%CI)	P	I^2^ (%)
rs920778																
Overall	12	**1.298(1.203-1.400)**	0.000	13.0	**2.289(1.815-2.887)**	0.000	54.3	**1.422(1.322-1.529)**	0.000	31.1	**2.028(1.641-2.506)**	0.000	57.9	**1.414(1.336-1.497)**	0.000	48.2
Caucasian	3	0.920(0.688-1.230)	0.574	0.0	1.296(0.875-1.920)	0.196	12.7	1.004(0.764-1.318)	0.978	0.0	1.368(0.960-1.949)	0.083	48.0	1.096(0.906-1.326)	0.343	0.0
Asian	9	**1.331(1.231-1.440)**	0.000	0.0	**2.558(2.170-3.016)**	0.000	34.1	**1.460(1.354-1.574)**	0.000	12.2	**2.214(1.774-2.762)**	0.000	56.0	**1.450(1.366-1.539)**	0.000	33.7
Estrogen-Dependent	3	1.233(0.995-1.528)	0.056	22.7	**2.397(1.684-3.412)**	0.000	0.0	**1.435(1.171-1.759)**	0.001	0.0	**1.969(1.291-3.005)**	0.002	63.1	**1.449(1.269-1.654)**	0.000	0.0
Digestive	5	**1.317(1.175-1.477)**	0.000	1.4	**2.566(1.766-3.727)**	0.000	57.1	**1.457(1.305-1.625)**	0.000	22.9	**2.274(1.594-3.244)**	0.000	56.2	**1.456(1.333-1.590)**	0.000	46.4
HB	4	**1.310(1.116-1.539)**	0.001	35.9	**2.237(1.422-3.518)**	0.000	55.7	**1.470(1.262-1.713)**	0.000	30.4	**2.090(1.380-3.167)**	0.001	54.1	**1.470(1.305-1.655)**	0.000	46.5
PB	5	**1.255(1.108-1.422)**	0.000	5.8	**2.260(1.486-3.436)**	0.000	62.8	**1.390(1.234-1.565)**	0.000	32.8	**1.969(1.367-2.837)**	0.000	69.8	**1.399(1.280-1.529)**	0.000	49.9
TaqMan	2	0.914(0.619-1.349)	0.651	0.0	1.475(0.893-2.436)	0.129	39.4	1.044(0.723-1.508)	0.818	0.0	1.580(0.716-3.487)	0.257	67.4	1.166(0.913-1.489)	0.218	0.0
RFLP	9	**1.314(1.211-1.425)**	0.000	11.6	**2.385(1.829-3.109)**	0.000	56.7	**1.431(1.324-1.548)**	0.000	38.1	**2.061(1.619-2.624)**	0.000	61.0	**1.425(1.300-1.562)**	0.000	52.9
rs4759314																
Overall	13	1.089(0.996-1.191)	0.063	47.1	1.084(0.735-1.599)	0.683	0.0	1.088(0.997-1.188)	0.059	47.7	1.087(0.738-1.602)	0.674	0.0	1.082(0.996-1.175)	0.062	47.4
Asian	12	**1.104(1.008-1.209)**	0.034	45.9	0.985(0.650-1.493)	0.943	0.0	**1.099(1.005-1.202)**	0.040	49.5	0.976(0.644-1.479)	0.908	0.0	1.092(0.964-1.237)	0.168	51.4
Digestive	8	1.082(0.904-1.296)	0.390	58.2	0.801(0.468-1.371)	0.419	0.0	1.076(0.897-1.290)	0.430	60.1	0.799(0.467-1.367)	0.413	0.0	1.065(0.895-1.266)	0.480	60.7
Estrogen-dependent	3	1.153(0.978-1.360)	0.091	8.5	1.416(0.696-2.883)	0.338	0.0	1.163(0.989-1.368)	0.068	20.3	1.387(0.682-2.820)	0.367	0.0	1.127(0.858-1.480)	0.057	27.6
PB	5	0.975(0.829-1.146)	0.759	0.0	0.892(0.424-1.878)	0.763	0.0	0.972(0.829-1.140)	0.726	0.0	0.903(0.430-1.899)	0.788	0.0	0.971(0.835-1.129)	0.699	0.0
HB	7	1.131(0.925-1.383)	0.231	66.6	1.177(0.736-1.880)	0.497	26.0	1.141(0.936-1.392)	0.191	66.9	1.176(0.737-1.877)	0.496	28.4	1.142(0.949-1.374)	0.159	67.0
TaqMan	6	1.149(0.916-1.441)	0.231	70.4	0.852(0.493-1.470)	0.565	27.1	1.142(0.905-1.442)	0.264	73.1	0.847(0.491-1.462)	0.551	25.4	1.126(0.898-1.412)	0.303	74.6
RFLP	5	1.047(0.887-1.236)	0.589	0.0	0.953(0.349-2.601)	0.925	0.0	1.044(0.886-1.231)	0.604	0.0	0.946(0.347-2.582)	0.914	0.0	1.040(0.887-1.218)	0.629	0.0
rs7958904																
Overall	6	**0.887(0.819-0.961)**	0.003	0.0	0.747(0.529-1.056)	0.098	79.2	**0.869(0.806-0.938)**	0.000	47.4	0.787(0.571-1.086)	0.145	77.0	**0.869(0.769-0.981)**	0.024	73.8
Estrogen-dependent	2	0.920(0.813-1.042)	0.191	43.0	0.910(0.312-2.653)	0.863	94.5	0.911(0.676-1.227)	0.538	84.0	0.944(0.345-2.577)	0.910	94.1	0.928(0.650-1.325)	0.682	92.6
Digestive	3	**0.870(0.776-0.975)**	0.017	0.0	**0.717(0.579-0.888)**	0.002	0.0	**0.842(0.756-0.939)**	0.002	0.0	**0.758(0.615-0.933)**	0.009	0.0	**0.852(0.781-0.930)**	0.000	0.0
HB	5	**0.899(0.823-0.982)**	0.018	0.0	0.804(0.552-1.172)	0.258	78.4	**0.891(0.819-0.969)**	0.007	48.3	0.845(0.596-1.198)	0.344	75.9	0.890(0.778-1.019)	0.091	73.4
TaqMan	4	**0.907(0.826-0.997)**	0.043	0.0	0.866(0.568-1.321)	0.503	81.1	0.897(0.782-1.028)	0.118	55.5	0.904(0.611-1.336)	0.611	79.0	0.912(0.783-1.062)	0.234	76.9
MALDI-TO F-MS	2	**0.838(0.720-0.975)**	0.022	0.0	**0.538(0.399-0.726)**	0.000	0.0	**0.783(0.678-0.905)**	0.001	0.0	**0.577(0.430-0.774)**	0.000	0.0	**0.779(0.693-0.876)**	0.000	0.0
rs1899663																
Overall	6	0.958(0.862-1.066)	0.431	0.0	0.810(0.596-1.101)	0.179	0.0	0.942(0.850-1.045)	0.260	0.0	0.818(0.608-1.100)	0.184	0.0	0.939(0.858-1.027)	0.166	0.0
Asian	5	0.950(0.852-1.058)	0.350	0.0	0.726(0.512-1.028)	0.071	0.0	0.932(0.839-1.036)	0.193	0.0	0.735(0.520-1.040)	0.082	0.0	0.925(0.842-1.016)	0.102	0.0
Estrogen-dependent	2	1.001(0.832-1.205)	0.991	0.0	0.746(0.430-1.293)	0.296	0.0	0.979(0.818-1.173)	0.822	18.0	0.752(0.435-1.301)	0.308	0.0	0.961(0.821-1.125)	0.620	23.1
Digestive	2	0.973(0.831-1.138)	0.729	0.0	0.773(0.463-1.290)	0.325	0.0	0.957(0.821-1.116)	0.576	0.0	0.775(0.465-1.292)	0.328	0.0	0.948(0.826-1.087)	0.443	0.0
HB	2	1.106(0.883-1.387)	0.381	0.0	1.080(0.638-1.828)	0.774	0.0	1.099(0.881-1.369)	0.403	0.0	1.022(0.632-1.651)	0.930	0.0	1.067(0.892-1.277)	0.475	0.0
PB	3	0.956(0.834-1.095)	0.512	0.0	0.737(0.487-1.114)	0.147	0.0	0.937(0.821-1.069)	0.334	0.0	0.746(0.494-1.125)	0.162	0.0	0.928(0.825-1.043)	0.208	0.0
RFLP	4	0.920(0.816-1.038)	0.176	0.0	0.701(0.480-1.024)	0.066	0.0	0.903(0.803-1.015)	0.086	0.0	0.715(0.491-1.043)	0.081	0.0	**0.899(0.810-0.997)**	0.044	0.0
rs87494																
Overall	3	1.100((0.989-1.223)	0.079	0.0	1.161(0.891-1.512)	0.269	0.0	1.106(0.999-1.225)	0.053	0.0	1.127(0.867-1.465)	0.372	0.0	**1.091((1.000-1.191)**	0.050	0.0
Digestive	2	1.092(0.955-1.250)	0.199	0.0	1.171(0.839-1.635)	0.353	0.0	1.101(0.967-1.253)	0.148	0.0	1.140(0.819-1.588)	0.436	0.0	1.090(0.975-1.218)	0.132	0.0
rs12826786																
Overall	5	1.155(0.971-1.374)	0.104	22.8	**1.670(1.244-2.242)**	0.001	48.2	**1.233((1.044-1.456)**	0.013	29.5	**1.551(1.186-2.027)**	0.001	40.5	**1.237(1.092-1.401)**	0.001	34.9
Caucasian	4	1.092(0.851-1.400)	0.489	36.9	1.507(0.903-2.515)	0.116	53.9	1.174(0.928-1.485)	0.181	43.2	**1.433(1.059-1.938)**	0.020	45.6	**1.196(1.017-1.406)**	0.030	47.8
Digestive	2	1.158(0.929-1.443)	0.191	0.0	1.595(0.796-3.196)	0.188	58.2	1.225(0.991-1.514)	0.060	14.4	**1.569(1.034-2.378)**	0.034	45.0	**1.224(1.034-1.448)**	0.019	29.1
HB	4	1.183(0.978-1.430)	0.084	37.9	**1.929(1.392-2.674)**	0.000	16.0	**1.289(1.074-1.546)**	0.006	31.4	**1.741(1.299-2.333)**	0.000	4.3	**1.299(1.134-1.489)**	0.000	2.1
TaqMan	2	0.875(0.597-1.283)	0.494	0.0	1.512(0.919-2.487)	0.104	47.3	1.018(0.711-1.456)	0.923	0.0	1.665(0.786-3.527)	0.183	62.4	1.169(0.914-1.495)	0.212	19.7
RFLP	2	1.169(0.947-1.443)	0.147	0.0	1.409(0.549-3.616)	0.476	75.8	1.211(0.989-1.484)	0.064	20.4	1.344(0.558-3.236)	0.510	73.8	1.145(0.856-1.532)	0.363	61.8

*OR* Odds ratio, *CI* Confidence interval. If P<0.05, the results are in bold. If I^2^>50%, the results were calculated by random model. *HB* Hospital-based, *PB* Population-based, *RFLP* restriction fragment length polymorphism, *MOLDI-TOF-MS* Matrix-Assisted Laser Desorption/Ionization Time of Flight Mass Spectrometry

For the rs12826786 C>T, this is first meta-analysis to evaluate the relationship in whole population by pooling 5 published studies comprising 1048 cases and 1432 controls. The results of the pooled analyses distinctly indicated that rs12826786 genetic variation was increased with susceptibility of cancer in recessive, dominant, allelic, and homozygous(TTvs.CC: OR = 1.670, 95% CI = 1.244–2.242, *I*^2^ = 48.20%; CT + TT vs. CC: OR = 1.233, 95% CI = 1.044–1.456, *I*^2^ = 29.50%; TT vs. CT + CC: OR = 1.551, 95% CI = 1.186–2.027, *I*^2^ = 40.50%; T vs. C: OR = 1.237, 95% CI = 1.092–1.401, *I*^2^ = 34.90%) models, and the associations were similar with the results of hospital-based control subgroup. Moreover, we reanalyze after excluded one study of non-satisfying HWE, the heterogeneity of the merged studies is not improved.

For the rs7958904 G>C, 6 eligible studies in Chinese population, totally consisting of 5123 cases and 5701 controls after excluded 1 study (Zhou-b) that genotype distribution of their controls did not conformed to HWE. Overall, we identified a significantly decreased susceptibility to overall cancer risk in all genetic (GC vs. GG: OR = 0.887, 95% CI = 0.819–0.961, *I*^2^ = 0%; CC + GC vs. GG: OR = 0.869, 95% CI = 0.806–0.938, *I*^2^ = 47.40%; C vs. G: OR = 0.869, 95% CI = 0.769–0.981, *I*^2^ = 73.8%) models rather than recessive and homozygous models, and a similar results was obtained in digestive cancers, hospital-based control and MALDI-TOF-MS subgroup. In addition, the heterogeneity of both digestive cancers and MALDI-TOF-MS subgroup was significantly lower than the other subgroups.

#### Rs4759314, rs1899663, and rs874945 polymorphism and cancer susceptibility

**Fig. 2:**
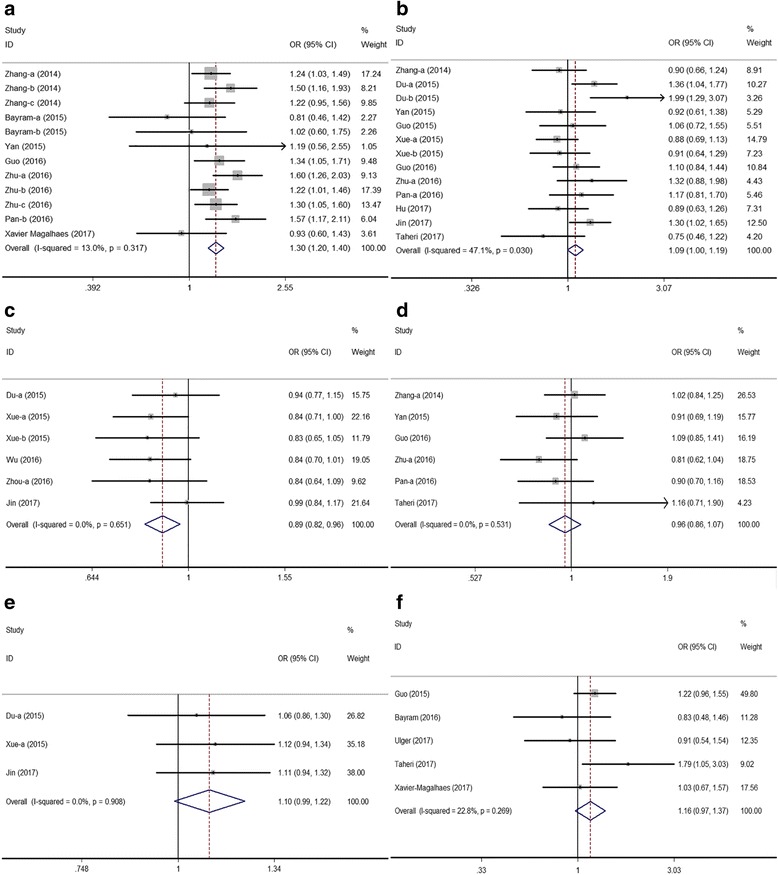
Forests plots for *HOTAIR* polymorphisms and cancer risk in heterozygous model. **a** rs920778, **b** rs4759314, **c** rs7958904, **d** rs1899663, **e** rs874945, **f** rs12826786

For the rs4759314 genetic variation, a total of 13 eligible case-control studies, comprising 8350 cases and 9940 controls were enrolled. For rs1899663 polymorphism, 6 eligible studies with 3239 cases and 4067 controls were included. For rs874945 genetic variation, 3 eligible studies consisted of 3069 cases and 3548 controls were included. Overall, the general OR with its 95% CI did not reveal a significant risk in all genetic models for rs4759314, rs1899663 and rs874945, and subsequent subgroup analyses also did not show the statistical associations rather than the significant risk of cancers correlated with rs4759314 polymorphism in heterozygous and dominant models for Chinese subgroup and rs874945 had a significantly increased risk of overall cancer was shown in allelic model. We detected that the heterogeneity of these subgroups (including estrogen-dependent cancers, RFLP, and population-based control group) was observed significantly decreased compared with other subgroups. In the study, Fig. 2 presented forest plots in heterozygous model for six common polymorphisms.

### Sensitivity analysis and publication bias

**Fig. 3:**
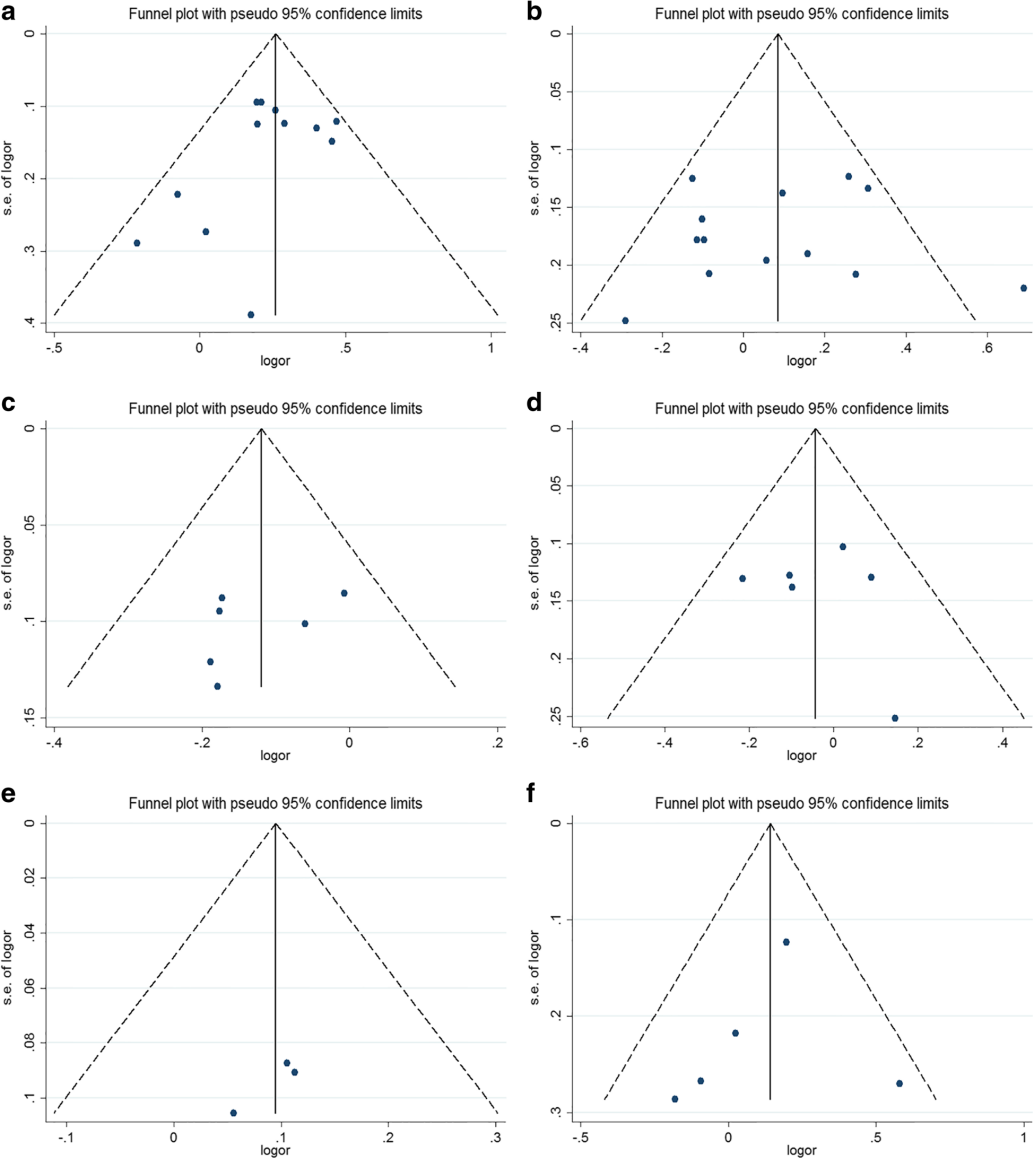
Funnel plots for *HOTAIR* polymorphisms and cancer risk in heterozygous model. **a** rs920778, **b** rs4759314, **c** rs7958904, **d** rs1899663, **e** rs874945, **f** rs12826786

Results of sensitivity analysis of six polymorphisms identified that any single study not qualitatively changed the all pooled ORs in five genetic models, which indicated that all the results of our meta-analysis remained robust and stable in both Asian and Caucasian population, the results of the heterozygous model are shown in Additional file 2: Figure S1. Then, we evaluated published bias by performing Begg’s funnel plots and Egger’s test. As illustrated in all funnel plots, the shapes of plots were no obvious asymmetry under dominant, recessive, allelic, heterozygous, and homozygous models for six genetic variations, and Fig. 3 only presented in heterozygous model. Additionally, all statistical results of Egger’s test illustrated the all corresponding *P* values of *t* test > 0.05, and the all relevant 95% CIs, including 0, were not listed. These statistical results further supported for the absence of publication bias in five genetic models, and so our results remained credibly and reliably.

## Discussion

Previously, there have been several meta-analysis [39–41] to pool eligible studies to examine the relationship between cancer risk and *HOTAIR* polymorphisms (including rs920778, rs4759314, rs7958904, rs874945, and rs1899663), and most of which included all studies and did not take into account the rejection of studies that the genotype distribution of controls did not conform to the HWE except Lv et al.’s study [42]. Among the 18 studies included in our updated meta-analysis, 12 include Asian populations and 6 include Caucasian populations. In addition, this is the first time to assess the effect of rs12826786 polymorphism on cancer susceptibility and also evaluate the relationships of abovementioned polymorphisms in *HOTAIR* with cancer risk. Overall, the results provided that rs920778, rs7958904, and rs12826786 but not rs4759314, rs1899663, and rs874945 loci are related to cancer risk among Caucasian and Asian populations, among of which rs920778 and rs12826786 increase and rs7958904 decreases cancer risk, respectively. To some extent, these findings denoted that the polymorphisms in *HOTAIR* may be related to the development of varieties of cancers and offered a novel and compelling evidence for functional analysis of the effects on susceptibility loci to diseases.

*HOTAIR*, a functional trans-acting lncRNA specifically transcribed from the *HOXC* gene, which is situated the antisense strand within the intergenic region between *HOXC11* and *HOXC12* on chromosome 12 [8, 43], and more and more studies focused on the mechanism of *HOTAIR*, and all these evidences have confirmed that *HOTAIR* expression lead to malignant transformation of normal cells in varieties of cancers such as ovarian cancers [44, 45], pancreatic tumors [10], hepatocellular carcinoma [11, 46–48], and ESCC [49, 50] to a certain extent. Various researches have previously demonstrated SNPs in several lncRNAs may be involved in carcinogenesis by influencing the secondary structure of the corresponding mRNA or altering its interacting partners [51, 52], which implied that functional susceptibility loci play a crucial role in occurrence and development of cancer. Therefore, we speculated polymorphisms in *HOTAIR* may also be related to cancer risk by modifying the secondary structure of *HOTAIR* then indirectly involved the relevant signaling pathways. Recently, several lines of published studies have researched the associations of *HOTAIR* polymorphisms with different cancers susceptibility, whereas their conclusions are discordant. Regarding the *HOTAIR* rs920778, Zhang et al. identified the SNP impacts *HOTAIR* expression via the gene intronic enhancer, which is located in between + 1719 bp and + 2353 bp from the transcriptional start site, with higher *HOTAIR* expression among T allele carriers, through reporter assays, which might be a potential genetic basis or mechanism for altering susceptibility of ESCC [21].These conclusions of Zhang et al. and other studies were in line with our results that the genetic variation of rs920778 increased cancer risk. But, our results showed the rs920778 SNP was significantly associated with an increased risk of cancer in Chinese rather than in Caucasian, according to the stratified analyses by ethnicity. The possible reasons for no statistically significant results in Caucasians are as follows. First, differences of two subgroups may be due to differences of genetic or inherited background in different population. For example, based on the dbSNP data from NCBI, the allele frequencies of the rs920778 polymorphisms are diverse between Chinese and Caucasian populations. Second, it is likely that there is not enough statistical power to obtain a convincing result in Caucasian population on the grounds that the sample size in Asian population (12,150n) is about 13 times the of Caucasian (934n). Finally, the different types of cancers as well as other unknown and uncontrollable factors may also be potential reasons for the differences of the findings between Asians and Caucasians, and differences of other subgroups maybe ascribe to the same reason mentioned above. Rs12826786 SNP, located on the promoter region of *HOTAIR* gene, Guo et al. reported that subjects carrying the rs12826786 TT genotype existed a remarkably elevated level of *HOTAIR* expression than those with the CC genotype in GCA tumor tissue [25], the results was consistent with our findings that rs12826786 polymorphism significantly increased susceptibility to cancer risk in all genetic models except heterozygous models, indicated that C-to-T transition of rs12826786 may first influence the transcription of *HOTAIR* then affecting the expression of the gene and ultimately impacts the susceptibility of various cancers. Rs7958904 genetic polymorphism, which is located on the exon of *HOTAIR* gene, we identified the SNP significantly decreased susceptibility to overall cancer risk in all genetic models rather than recessive and homozygous models. Jin et al. also obtained that the rs7958904 CC genotype increased risk of cervical cancer compared with the GG genotypes on the grounds of functional assays, which showed patients with rs7958904 CC genotype existed higher *HOTAIR* expression than with GG genotype [27]. Additionally, Xue et al. revealed that rs7958904 G/C variation strikingly altered the secondary structure by silico analyses, which indicated that the genetic variation may participate in influencing the susceptibility of colorectal cancer through changing for *HOTAIR* structure [22]. Besides, Taheri et al. and his colleagues found that rs1899663 and rs12826786 may alter the affinity for binding of some relevant transcription factors, which be related to the occurrence and progression of various cancers including prostate cancer [37]. Moreover, Du et al. attained that *HOTAIR* and *HOXC11* expression levels were higher in subjects with the rs4759314 AG genotype than with AA genotype in GC tissues. Simultaneously, they further explored the SNP, in an intronic promoter region, influenced the activity of this promoter and contributed to the expression of *HOTAIR* and its downstream gene *HOXC11* in a genotype-specific way by The Cancer Genome Atlas (TCGA) database, which is an underlying mechanism for GC susceptibility [29]. However, our meta-analysis found that rs1899663 (intron), rs8749459 (3′ near gene), and rs4759314 polymorphisms were not significantly associated with susceptibility to cancer. Therefore, it is possible that genetic polymorphisms as inherited basis that may modify the function or expression of the involved genes, ultimately contribute to cancer risk based on the above evidence.

In the present meta-analysis, there existed a few limitations potentially. First, the study presented latent language bias due to all published studies only restricted in English. Second, analysis of stratification resulted in smaller sample size in the subgroup that affected statistical efficacy. For example, compared to Caucasian population, most of the studies included in the meta-analysis focused on Chinese population to estimate *HOTAIR* polymorphisms how to contribute to cancer susceptibility, and therefore need to broaden the sample size in the Caucasian to further verify how *HOTAIR* polymorphism affects the susceptibility of the associated cancer. Third, we cannot further calculate gene-environment interactions or cumulative effects of genetic polymorphisms on account of not getting original data. Fourth, after excluding those that did not follow the HWE, the sample size of the included studies is too small for rs874945 polymorphism. Fifth, the adjustment of confounders between the original studies is inconsistent, and the study also cannot unify the adjustment of confounding factors (including age, gender, smoking, drinking, etc.) for all included case-control studies due to not obtaining detailed raw data. Finally, due to most of the original publications did not do gender-based stratification analysis, the study failed to perform subgroup analyses of gender. These deficiencies may fail to correctly reveal how *HOTAIR* polymorphisms affect the risk of cancer. Thus, pooled results of the presented comprehensive analyses should be elucidated cautiously.

## Conclusions

The meta-analysis provided that three functional polymorphisms of *HOTAIR* with rs920778, rs7958904, and rs12826786 might contribute to genetic susceptibility to cancer risk in overall population, whereas rs1899663, rs4759314, and rs874945 had no significant associations. In consequence, well-conducted studies with sufficient sample size are necessarily demanded to further test the association of the abovementioned polymorphisms in *HOTAIR* and cancer risk, especially in Caucasian and other types of cancers.

## Additional files

Additional file 1: **Table S1.** Genotype frequency distributions of HOTAIR polymorphisms and cancer risk. (DOCX 38 kb)

Additional file 2: **Figure S1.** Sensitivity analyses for *HOTAIR* polymorphisms and cancer risk in heterozygous model. (A) rs920778, (B) rs4759314, (C) rs7958904, (D) rs1899663, (E) rs874945, and (F) rs12826786. (JPEG 754 kb)

## Abbreviations

ceRNA: Competing endogenous RNAs
HOTAIR: HOX transcript anti-sense RNA
PRC2: Polycomb repressive complex 2
