# Supplementary material for: Long non-coding RNA HOTAIR polymorphism and susceptibility to cancer: an updated meta-analysis
Source: Environ Health Prev Med. 2018 Feb 20;23:8. doi: 10.1186/s12199-018-0697-0 (PMC5819648; doi:10.1186/s12199-018-0697-0)
Supplement: Supplementary file 1 — Table S1. Genotype frequency distributions of HOTAIR polymorphisms and cancer risk. (DOCX 38 kb) [file 12199_2018_697_MOESM1_ESM.docx]

**Table S1** Genotype frequency distributions of HOTAIR polymorphisms and cancer risk.

| **First author** | **Year** | **country** | **ethnicity** | | **Source of control** | **Genotyping methods** | | **Type of cancers** | **Case** | **Control** | **Genotype distribution** | | | | | | ***P* for HWE** | **Included in meta-analysis** |
| --- | --- | --- | --- | --- | --- | --- | --- | --- | --- | --- | --- | --- | --- | --- | --- | --- | --- | --- |
|  |  |  |  |  |  |  |  |  |  |  | **Case** | | | **Control** | | |  |  |
| rs920778 C＞T | | | | | | | | | | | CC | TC | TT | CC | TC | TT |  |  |
| Zhang-a[21] | 2014 | china | Asian | PB | | | RFLP | ESCC | 1000 | 1000 | 528 | 389 | 83 | 601 | 358 | 41 | 0.173 | Yes |
| Zhang-b[21] | 2014 | china | Asian | HB | | | RFLP | ESCC | 510 | 550 | 256 | 207 | 47 | 344 | 186 | 20 | 0.401 | Yes |
| Zhang-c[21] | 2014 | china | Asian | PB | | | RFLP | ESCC | 588 | 600 | 307 | 203 | 51 | 378 | 205 | 17 | 0.082 | Yes |
| Bayram-a[33] | 2015 | Turkey | Caucasian | HB | | | TaqMan | BC | 123 | 122 | 40 | 52 | 31 | 41 | 66 | 15 | 0.140 | Yes |
| Bayram-b[28] | 2015 | Turkey | Caucasian | HB | | | TaqMan | GC | 104 | 209 | 32 | 52 | 20 | 66 | 105 | 38 | 0.738 | Yes |
| Yan[31] | 2015 | china | Asian | PB | | | RFLP | BC | 502 | 504 | 12 | 151 | 339 | 18 | 190 | 296 | 0.060 | Yes |
| Guo[32] | 2016 | china | Asian | HB | | | MALDI-TOF-MS | CC | 510 | 713 | 269 | 189 | 52 | 448 | 235 | 30 | 0.907 | Yes |
| Zhu-a[34] | 2016 | china | Asian | NA | | | RFLP | PTC | 600 | 600 | 288 | 259 | 53 | 372 | 209 | 19 | 0.109 | Yes |
| Zhu-b[34] | 2016 | china | Asian | NA | | | RFLP | PTC | 1000 | 1000 | 553 | 385 | 62 | 608 | 348 | 44 | 0.513 | Yes |
| Zhu-c[34] | 2016 | china | Asian | NA | | | RFLP | PTC | 800 | 800 | 416 | 316 | 68 | 485 | 284 | 31 | 0.181 | Yes |
| Qiu-a[18] | 2016 | china | Asian | HB | | | Taqman | Ovarian cancer | 190 | 380 | 138 | 37 | 15 | 321 | 48 | 11 | 0.000 | No |
| Qiu-b[18] | 2016 | china | Asian | HB | | | Taqman | Ovarian cancer | 139 | 300 | 97 | 32 | 10 | 259 | 30 | 11 | 0.000 | No |
| Qiu[24] | 2016 | china | Asian | NA | | | Taqman | CC | 215 | 430 | 90 | 78 | 47 | 226 | 150 | 54 | 0.000 | No |
| Pan-a[26] | 2016 | china | Asian | PB | | | RFLP | GC | 500 | 1000 | 275 | 194 | 31 | 608 | 368 | 24 | 0.000 | No |
| Pan-b[26] | 2016 | china | Asian | PB | | | RFLP | GC | 300 | 600 | 145 | 127 | 28 | 372 | 207 | 21 | 0.230 | Yes |
| Xavier‑Magalhaes[35] | 2017 | Portugal | Caucasian | PB | | | RFLP | Glioma | 177 | 199 | 82 | 71 | 24 | 90 | 84 | 25 | 0.438 | Yes |
| rs4759314 A＞G | | | | | | | | | | | AA | GA | GG | AA | GA | GG |  |  |
| Zhang-a[21] | 2014 | china | Asian | | PB | RFLP | | ESCC | 1000 | 1000 | 917 | 81 | 2 | 910 | 89 | 1 | 0.436 | Yes |
| Du-a[29] | 2015 | china | Asian | | HB | Taqman | | GC | 753 | 1057 | 624 | 126 | 3 | 915 | 136 | 6 | 0.699 | Yes |
| Du-b[29] | 2015 | china | Asian | | HB | Taqman | | GC | 522 | 589 | 459 | 60 | 3 | 549 | 36 | 2 | 0.098 | Yes |
| Yan[31] | 2015 | china | Asian | | PB | RFLP | | BC | 502 | 504 | 451 | 50 | 1 | 448 | 54 | 2 | 0.785 | Yes |
| Guo[25] | 2015 | china | Asian | | PB | RFLP | | GCA | 515 | 654 | 461 | 53 | 1 | 589 | 64 | 1 | 0.587 | Yes |
| Xue-a[22] | 2015 | china | Asian | | HB | Taqman | | CRC | 1147 | 1203 | 1011 | 135 | 1 | 1037 | 157 | 9 | 0.260 | Yes |
| Xue-b[22] | 2015 | china | Asian | | HB | Taqman | | CRC | 587 | 652 | 517 | 65 | 4 | 571 | 79 | 2 | 0.673 | Yes |
| Guo[32] | 2016 | china | Asian | | HB | MALDI-TOF-MS | | CC | 510 | 713 | 378 | 121 | 11 | 544 | 158 | 11 | 0.903 | Yes |
| Zhu-a[34] | 2016 | china | Asian | | NA | RFLP | | PTC | 600 | 600 | 540 | 58 | 2 | 553 | 45 | 2 | 0.297 | Yes |
| Wu[30] | 2016 | china | Asian | | NA | MALDI-TOF-MS | | EOC | 1000 | 1000 | 819 | 140 | 41 | 852 | 125 | 23 | 0.000 | No |
| Zhou-a[38] | 2016 | china | Asian | | HB | MALDI-TOF-MS | | Osteosarcoma | 500 | 500 | 423 | 62 | 15 | 425 | 64 | 11 | 0.000 | No |
| Pan-a[26] | 2016 | china | Asian | | PB | RFLP | | GC | 500 | 1000 | 451 | 48 | 1 | 914 | 83 | 3 | 0.448 | Yes |
| Hu[36] | 2017 | china | Asian | | PB | TaqMan | | Pancreatic cancer | 416 | 416 | 333 | 75 | 8 | 325 | 82 | 9 | 0.166 | Yes |
| Jin[27] | 2017 | china | Asian | | HB | TaqMan | | CC | 1174 | 1304 | 1012 | 158 | 4 | 1162 | 140 | 2 | 0.294 | Yes |
| Taheri[37] | 2017 | Iran | Caucasian | | HB | ARMS-PCR | | Prostate cancer | 125 | 250 | 86 | 32 | 7 | 163 | 81 | 6 | 0.268 | Yes |
| rs7958904 G＞C | | | | | | | | | | | GG | CG | CC | GG | CG | CC |  |  |
| Du-a[29] | 2015 | china | Asian | | HB | Taqman | | GC | 753 | 1057 | 412 | 276 | 51 | 568 | 404 | 85 | 0.271 | Yes |
| Xue-a[22] | 2015 | china | Asian | | HB | Taqman | | CRC | 1147 | 1203 | 672 | 399 | 74 | 646 | 456 | 99 | 0.147 | Yes |
| Xue-b[22] | 2015 | china | Asian | | HB | Taqman | | CRC | 587 | 652 | 347 | 206 | 33 | 346 | 248 | 57 | 0.192 | Yes |
| Wu[30] | 2016 | china | Asian | | NA | MALDI-TOF-MS | | EOC | 1000 | 1000 | 594 | 355 | 51 | 533 | 380 | 87 | 0.105 | Yes |
| Zhou-a[38] | 2016 | china | Asian | | HB | MALDI-TOF-MS | | Osteosarcoma | 500 | 500 | 295 | 180 | 25 | 266 | 194 | 40 | 0.580 | Yes |
| Zhou-b[38] | 2016 | china | Asian | | HB | MALDI-TOF-MS | | Osteosarcoma | 400 | 400 | 229 | 140 | 31 | 200 | 152 | 48 | 0.025 | No |
| Jin[27] | 2017 | china | Asian | | HB | TaqMan | | CC | 1153 | 1292 | 640 | 427 | 86 | 735 | 494 | 63 | 0.083 | Yes |
| rs874945 G＞A | | | | | | | | | | | GG | AG | AA | GG | AG | AA |  |  |
| Du-a[29] | 2015 | china | Asian | | HB | Taqman | | GC | 753 | 1057 | 495 | 225 | 31 | 714 | 307 | 36 | 0.672 | Yes |
| Xue-a[22] | 2015 | china | Asian | | HB | Taqman | | CRC | 1147 | 1203 | 751 | 356 | 40 | 817 | 346 | 39 | 0.749 | Yes |
| Wu[30] | 2016 | china | Asian | | NA | MALDI-TOF-MS | | EOC | 1000 | 1000 | 665 | 283 | 52 | 677 | 279 | 44 | 0.029 | No |
| Zhou-a[38] | 2016 | china | Asian | | HB | MALDI-TOF-MS | | Osteosarcoma | 500 | 500 | 310 | 150 | 40 | 338 | 135 | 27 | 0.008 | No |
| Zhou-b[38] | 2016 | china | Asian | | HB | MALDI-TOF-MS | | Osteosarcoma | 400 | 400 | 267 | 106 | 27 | 270 | 108 | 22 | 0.014 | No |
| Jin[27] | 2017 | china | Asian | | HB | TaqMan | | CC | 1171 | 1289 | 745 | 383 | 43 | 852 | 394 | 43 | 0.757 | Yes |
| rs1899663 G＞T | | | | | | | | | | | GG | TG | TT | GG | TG | TT |  |  |
| Zhang-a[21] | 2014 | china | Asian | | PB | RFLP | | ESCC | 1000 | 1000 | 725 | 256 | 19 | 724 | 250 | 26 | 0.430 | Yes |
| Yan[31] | 2015 | china | Asian | | PB | RFLP | | BC | 502 | 504 | 339 | 149 | 14 | 326 | 158 | 20 | 0.876 | Yes |
| Guo[32] | 2016 | china | Asian | | HB | MALDI-TOF-MS | | CC | 510 | 713 | 356 | 146 | 8 | 509 | 191 | 13 | 0.308 | Yes |
| Zhu-a[34] | 2016 | china | Asian | | NA | RFLP | | PTC | 600 | 600 | 442 | 151 | 7 | 413 | 175 | 12 | 0.184 | Yes |
| Pan-a[26] | 2016 | china | Asian | | PB | RFLP | | GC | 500 | 1000 | 376 | 118 | 6 | 732 | 255 | 13 | 0.078 | Yes |
| Taheri[37] | 2017 | Iran | Caucasian | | HB | ARMS-PCR | | Prostate cancer | 127 | 250 | 35 | 70 | 22 | 77 | 133 | 40 | 0.165 | Yes |
| rs12826786 C>T |  |  |  | |  |  | |  |  |  | CC | TC | TT | CC | TC | TT |  |  |
| Guo[25] | 2015 | China | Asian | | HB | RFLP | | GCA | 515 | 654 | 285 | 200 | 30 | 403 | 232 | 19 | [0.034](file:///C:\Users\Administrator\Desktop\原始meta.xlsx#RANGE!_bookmark4) | Yes |
| Bayram[19] | 2016 | Turkey | Caucasian | | HB | TaqMan | | BC | 123 | 122 | 42 | 51 | 30 | 44 | 64 | 14 | [0.197](file:///C:\Users\Administrator\Desktop\原始meta.xlsx#RANGE!_bookmark5) | Yes |
| Ulger[23] | 2017 | Turkey | Caucasian | | HB | TaqMan | | GC | 105 | 207 | 38 | 47 | 20 | 73 | 99 | 35 | 0.884 | Yes |
| Taheri[37] | 2017 | Iran | Caucasian | | HB | ARMS-PCR | | Prostate cancer | 128 | 250 | 26 | 70 | 32 | 83 | 125 | 42 | 0.662 | Yes |
| Xavier‑Magalhaes[35] | 2017 | Portugal | Caucasian | | PB | RFLP | | Glioma | 177 | 199 | 84 | 77 | 16 | 94 | 84 | 21 | 0.730 | Yes |
| rs10783618 T>C |  |  |  | |  |  | |  |  |  | TT | TC | CC | TT | TC | CC |  |  |
| Guo[25] | 2015 | China | Asian | | HB | RFLP | | GCA | 515 | 654 | 262 | 212 | 41 | 365 | 249 | 40 | [0.775](file:///C:\Users\Administrator\Desktop\原始meta.xlsx#RANGE!_bookmark5) | NO |

BC=Breast cancer; CRC= Colorectal cancer; CC=Cervical cancer; ESCC=Esophageal squamous cell carcinoma; GC= Gastric cancer; GCA=Gastric cardia adenocarcinoma; PTC=Papillary thyroid carcinoma; HB=Hospital-based; PB=Population-based; HWE=Hardy-Weinberg equilibrium; RFLP=restriction fragment length polymorphism; MOLDI-TOF-MS=Matrix-Assisted Laser Desorption/ Ionization Time of Flight Mass Spectrometry; Bayram-a, b=BC, CC; Zhang-a, b & c=Jinan, Shijiazhuang & Huaian; Zhou-a & b=Stage1 & Stage2;Zhu-a,b & c=Shandong, Jiangsu & Jilin; Qiu-a, b=Zhengzhou, Wuxi; Pan-a, b=Jinan, Huaian; Du-a, b=Test, validation Set; Xue-a, b=Stage1, Stage2.
