# Supplementary figures and images for: Long non-coding RNA HOTAIR polymorphism and susceptibility to cancer: an updated meta-analysis
Source: Environ Health Prev Med. 2018 Feb 20;23:8. doi: 10.1186/s12199-018-0697-0 (PMC5819648; doi:10.1186/s12199-018-0697-0)

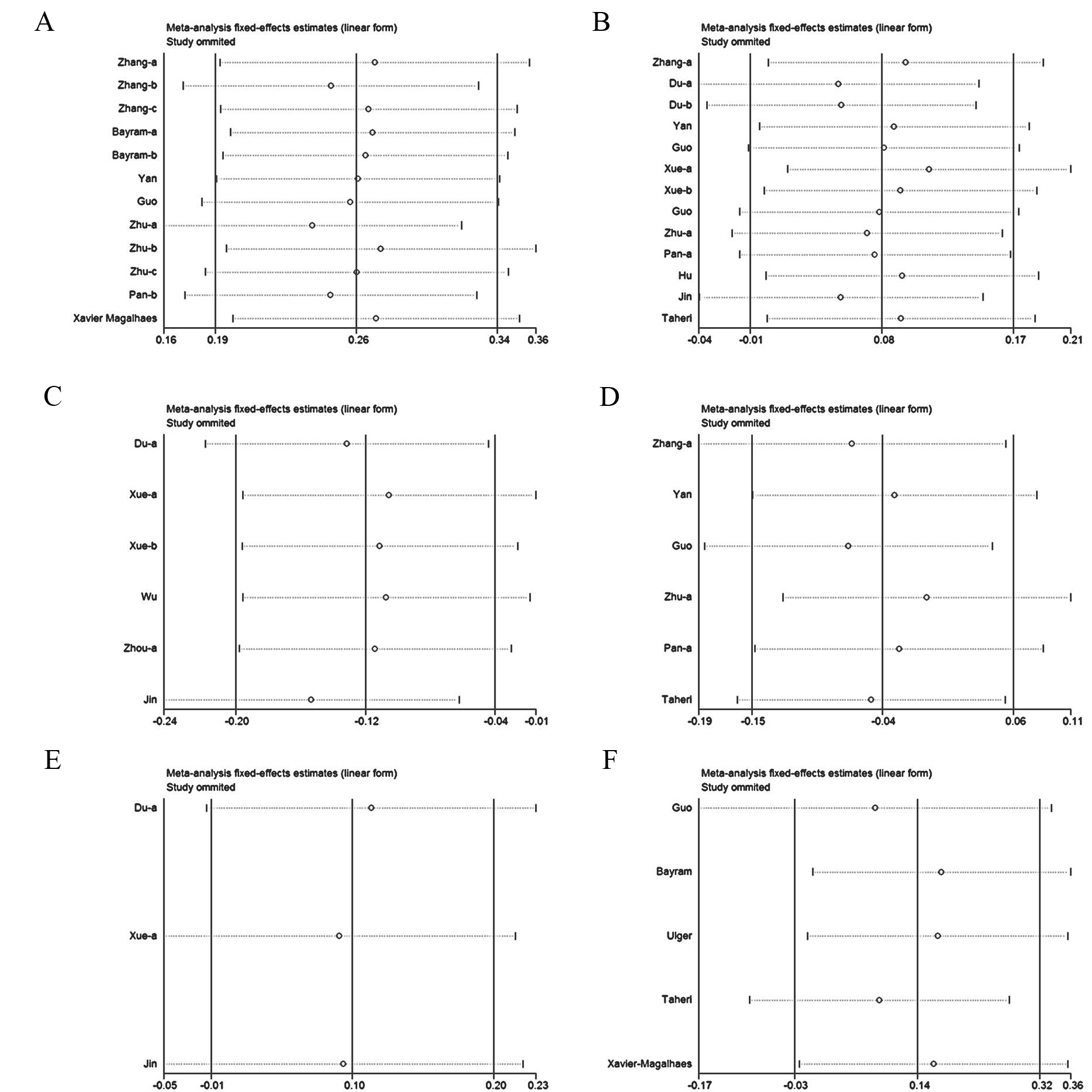

Supplement: Supplementary file 2 — Figure S1. Sensitivity analyses for HOTAIR polymorphisms and cancer risk in heterozygous model. (A) rs920778, (B) rs4759314, (C) rs7958904, (D) rs1899663, (E) rs874945, and (F) rs12826786. (JPEG 754 kb) [file 12199_2018_697_MOESM2_ESM.jpg]
